# Practices and Attitudes of Dental Loupes and Their Relationship to Musculoskeletal Disorders among Dental Practitioners

**DOI:** 10.1155/2020/8828709

**Published:** 2020-07-30

**Authors:** Khalid Aboalshamat, Ola Daoud, Lina Ayman Mahmoud, Sakina Attal, Rahaf Alshehri, Duaa Bin Othman, Reem Alzahrani

**Affiliations:** ^1^Dental Public Health Division, Preventative Dentistry Department, College of Dentistry, Umm Al-Qura University, Makkah, Saudi Arabia; ^2^Medicine and Medical Science Research Center, Deanship of Scientific Research, Umm Al-Qura University, Makkah, Saudi Arabia; ^3^Alfarabi College, Jeddah, Saudi Arabia

## Abstract

**Objective:**

A dental loupe is a tool used by many dentists and dental students to improve visual field and performance. This study aims to assess the practices and attitudes about dental magnification loupes and their relationship to musculoskeletal disorders.

**Materials and Methods:**

A cross-sectional study was conducted involving 400 dental students and dentists in four dental colleges in Jeddah, Saudi Arabia. The mean age was 28.35 years (SD = 8.50), and 56% of the participants were male. Also, 70.75% were students or interns, while 29.25% were dentists. Data gathering was conducted using a questionnaire that was composed of four sections: demographic, magnification devices usage, attitude toward dental loupes, and the Nordic questionnaire to assess musculoskeletal disorders.

**Results:**

A total of 66% of participants had used dental magnification tools before, but only 12.25% were using dental loupes at the time of the study. The main reported advantages of dental loupes were comfort in vision (59.25%) and improved work accuracy (53%), while the main disadvantage was difficulty in visual measurement (28.5%). A total of 73.25% reported that price was the main barrier to the use of dental loupes. Lower back (63.5%), neck (65.25%), and shoulder (46.25%) pain were the most frequently reported areas of discomfort. There was a significant relationship (<0.05) between the use of dental loupes and lowered levels of reported discomfort in the lower back, neck, shoulders, elbows, upper back, and feet.

**Conclusion:**

There are few dental professionals who use dental loupes in Saudi Arabia. There was a significant relationship between dental loupes use and reduction of musculoskeletal disorders among dental students and dentists.

## 1. Introduction

Dental magnification loupes are a tool used by dentists and dental hygienists to enhance their ability to visualize what cannot be seen by the naked eye [1]. Loupes, preceded by the dental operating microscope, were invented in the 1980s to increase success rates for surgical endodontic treatment [2]. Loupes are usually attached to glasses frames using different mounting formats, including fully adjustable front lens loupe, limited adjustable lens loupe, and through-the-lens loupes [3, 4]. Each one of these types has different abilities in terms of adjustment and ease of use. In addition, loupes are available with different magnification strengths [5].

 Several studies have reported that the use of loupes among dental students [6–8], dentists [9, 10], and even dental hygienists [11, 12] has become popular in many countries around the world. Magnification in dentistry was promoted to be used for oral surgical flaps, dental tissue grafts, surgical periodontal treatments [13], various steps to endodontic treatment [14, 15], caries detection, and cavity preparation [16]. Studies found many advantages to using loupes, such as decreased work time, increased work quality [7, 8], and easier detection of unfound canals [14].

More importantly, a systematic review pointed out that using loupes was found to be beneficial for reducing musculoskeletal disorders (MSDs) that are common among dental professionals, especially those in the hands, arms, and shoulders [17]. The study found little evidence, however, that loupes were effective for easing neck pain. Other articles indicated the importance of using loupes for dental professionals over 40 years old who have accentuated MSD problems due to visual deficiencies from increasing age [18]. This is important as the prevalence of MSDs is high among students, as has been reported in Saudi literature (43% to 95.8%) [19], and also among dentists, as indicated by another systematic review (64% to 93%) [20], and studies in Saudi Arabia (85% to 90.2%) [21, 22]. A study in Saudi Arabia found that the majority (70%) of dental students are ignorant about dental ergonomics, and half of them were aware of MSD. Also, most of them were unaware of MSDs prevention of treatment modalities [23]. In fact, MSDs are found to be a major contributor to early retirement and poor quality of life [20], which justifies the importance of loupes as an aid to alleviate this problem.

However, many articles pointed out that the advantages with loupes can best be fulfilled when proper knowledge and skills are acquired for their use [8, 24], especially during the early years of undergraduate dental study [6, 10, 25]. However, few studies have assessed levels of knowledge, attitudes, and practices using loupes and magnification devices among dentists and dental students. Studies have reported that 21.9% and 28% of dental students use loupes in, respectively, Saudi Arabia [6] and the UK [9], which are lower than the reported percentages of dentists using loupes in the UK (44%) [9], or the United States (56%) [10]. In fact, the most commonly used magnification tool among students was dental loupes, followed by magnification lenses [6]. Also, around 91.6% of students advocated for its effectiveness at improving work quality [6, 9]. The favored loupes were 2.5 times magnification and through-the-lens types [9]. Price was the most common barrier to the use of loupes according to some studies, in addition to a lack of experience [9, 25]. It should be noted that there is a proportion of dental faculty members who believe that loupes are not very important for undergraduate students [10], while some dental students believe that using loupes has disadvantages in that dentists might rely on loupes in their future dental practice [6].

There are very few studies that have assessed the use of dental loupes in Saudi Arabia and the relationship of loupe use to MSDs. Thus, this study aimed to assess usage of and attitudes about dental magnification loupes and the relationship of loupe use to MSDs among dental students and dentists in Jeddah, Saudi Arabia.

## 2. Materials and Methods

Participants were recruited using convenient sampling in this cross-sectional study. Study inclusion criteria were either faculty members, interns, or students at clinical levels (fourth, fifth, and sixth years) who were currently studying or on the job in dental colleges. Participants who did not sign the study consent were excluded. Data were collected from November to December 2019 from four dental colleges: Alfarabi Medical College (FMC), Ibn Sina National College (ISNC), Batterjee Medical College (BMC), and King Abdulaziz University (KAAU). The minimum required sample size was calculated using an expected prevalence of 50%, a confidence interval of 95%, and an alpha level of 5%, which resulted in 385 participants. To overcome an expected nonresponse rate of 30%, 500 participants were invited to answer the study's hard copy self-reported questionnaire. Participants answered the study questionnaire during breaks and free time, and all participants signed the study consent form before completing the questionnaire. All answers were taken anonymously, although the research team contacted study participants face to face. The time to answer the questionnaire was three to five minutes.

The questionnaire was composed of four sections as shown in Table 1. The questions in sections two and three were derived from previous studies, with modifications [6, 9, 25]. Section four included the Nordic work-related MSD assessment [26], which is a well-validated questionnaire [27–30] that has been used for decades. In addition, the questionnaire as a whole was tested in a pilot test with 10 participants not included in the main study assessing validation, especially for sections two and three in terms of content, organization, language, syntax, and logical flow.

Data analysis was conducted using SPSS v.21 (IBM Corp., Armonk, NY, USA). Data were analyzed using multiple logistic regression and chi-square tests and were presented as descriptive statistics by the mean, standard deviation, frequency, and percentages. A *P* value of less than 0.05 was considered significant. The study was approved by the Institutional Review Board of Umm Al-Qura University, Faculty of Dentistry, with number 154-19.

## 3. Results

A total of 400 dental students and dentists participated in this study, for a response rate of 80%, with a mean age of 28.35 years and standard deviation (SD) of 8.50. Participants' mean years in practice was 3.26 (SD = 7.29). Of the participants, 283 (70.75%) were students or interns, while 117 (29.25%) were dentists. Participant demographic data are shown in Table 2.

A total of 268 (67%) respondents had used magnification tools in dentistry in the past. Some of them used different devices simultaneously while others used only one at a time, and 240 (60%) used dental loupes, 15 (3.75%) used a magnifying glass, and 31 (7.75%) used a dental microscope. Among the current users of these tools, only 49 participants (12.25%) used dental loupes in their clinical practice. The multiple logistic regression (backward elimination technique) showed that none of the demographic variables had a significant relationship with current use of dental loupes. In other words, age, years of education, gender, educational level, and college type were not significantly related to the use of dental loupes.

**Figure 1:**
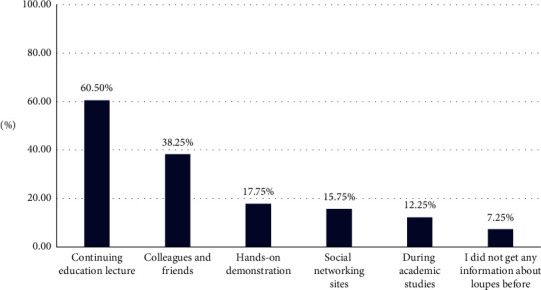
Participants' sources of information about dental loupes.

Participants had received information about dental loupes from different resources, and sometimes from multiple resources, which is explained in Figure 1. Participants reported different advantages and disadvantages to using dental loupes, and some participants reported more advantages than disadvantages. This is illustrated in Table 3. Participants also reported different barriers to using dental loupes when participants were able to report more than one barrier, as shown in Table 4.

**Figure 2:**
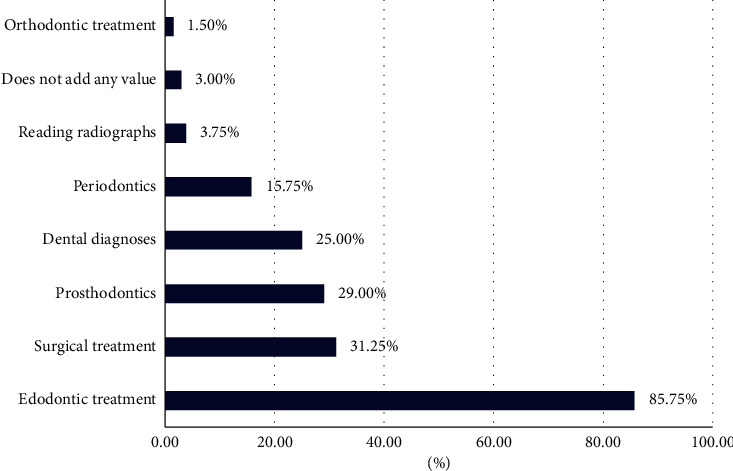
Dental specialties for which dental loupes were believed to be most useful.

Participants varied in their opinions regarding the use of dental loupes in different dental specialties, with some participants believing loupes can be used in more than specialty, as shown in Figure 2.

Participants reported different levels of musculoskeletal pain in various parts of their body and pain experienced during the prior seven days, as shown in Table 5. Also, using chi-square test and Fisher's exact test, current users of dental loupes were found to have significantly less trouble in their lower back, neck, shoulders, elbows, upper back, and feet, as shown in Table 6.

## 4. Discussion

This study aimed to assess the use of and attitudes toward dental magnification loupes and their relationship to MSDs among dental students and dentists. Two-thirds of the participants had used dental magnification tools before, and around one in every 10 was currently using dental loupes in a dental practice. The majority believed that dental loupes improve visual abilities and dental performance, while some others claimed that dental loupes cause visual measurement problems or pain in the neck and shoulders. Price was the main barrier to the use of dental loupes. While dental loupes have been associated with lowered levels of MSDs in most parts of the body, around half of the participants had MSDs in the neck, shoulder, or lower back.

Despite the fact that 60% of the participants in this study had used dental loupes before, the percentage of current users was low at 12.3%. In fact, this proportion is less than has been found in similar studies in the United Kingdom (27.7% to 44% among faculty members and students) [9] and the United States (53.7% among faculty members) [10]. This also was lower than a similar local study in Saudi Arabia (21.9% among students and residents) [6]. This might be because previous studies [6, 10, 31] assessed current users of various types of magnification all together and did not restrict the assessment to only dental loupes, such as was done in this study. Furthermore, the previous study in Saudi Arabia was conducted in 2017, and since that time, there has been an increase in taxes for some items in Saudi Arabia accompanied by a reform phase of Vision 2030 [32], so buying dental loupes might be seen as more a luxury than a necessity because it is not mandatory to use them in dental faculties in Saudi Arabia [6]. This seems to be supported by 73.25% of participants stating that the cost of dental loupes was the main barrier to their use, which will be discussed in later sections.

Most of the participants agreed that using dental loupes was most important in endodontic and surgical treatment, which is similar to previous studies [6, 25]. Around half of the participants in this study believed that dental loupes are helpful for vision comfort, improve work accuracy, and enhance the quality of treatment, which was similar to findings from other studies [7, 8]. Also, around one-third believed that dental loupes have no disadvantages, while among those who reported disadvantages, the most frequently cited disadvantage was difficulty in visual measuring. Also, both this study and the previous Saudi study [6] highlighted that a proportion of respondents did not want to become reliant on dental loupes in their work and considered that to be a disadvantage. This can perhaps be a result of improper use, as previous studies have found that the advantages of using dental loupes can only be tangible after proper training [8, 24].

Expense was the main barrier reported by three-fourths of the participants not using dental loupes, which is supported by previous studies in the UK and India [9, 25]. This might raise another recommendation of subsidizing the purchase of dental loupes for dental students, which could perhaps be accomplished by buying loupes in bulk for dental faculties in Saudi Arabia. Another method is to make dental loupes available during dental training so that dental students can acknowledge their importance in reducing the MSDs that have been found to be one of the primary causes of early retirement from dentistry [20].

In this study, the relationship between dental loupe use and MSDs was assessed in two ways, according to participants' attitudes and perceptions and by using statistical tests. First, for perceptions, one-fourth of the participants believed that dental loupes reduce MSDs symptoms, while 10.25% to 22.75% believed dental loupes increase MSDs. This controversial belief among participants cannot be explained, as the statistical assessment, as will be explained below, shows that using dental loupes is associated with a lowered incidence of MSDs. This finding might indicate that some dental students and dentists have misconceptions about dental loupes.

Statistically, the use of dental loupes is tied to a significantly lower prevalence of pain in the lower back, neck, shoulders, elbows, upper back, and feet. This is supported by a systematic review [17] that found dental loupes to be effective in reducing pain in the arms, hands, and neck. In fact, another Saudi study found that using dental loupes was the only ergonomic practice that was associated with a reduction in MSDs [33]. However, a recent study in the United States on the effect of mandatory dental loupe use for dental hygienists showed that the occurrence of MSDs remained the same [34]. This difference might be because the nature of the work conducted by dentists and dental hygienists is different. Regardless, our results are aligned with previous studies in indicating the importance of dental loupes as a valuable way to reduce the incidence of MSDs.

The data from this study show that the greatest percentage of sources of information about dental loupes among participants was from continuing education lectures, while a low proportion came from academic studies in universities. This is similar to the previous local study [6], which highlighted that dental loupes had not been formally taught in academic lectures. However, this finding is contradictory to a study in India, where 59.5% had received their information about loupes during their degree studies [25]. It seems that adding dental loupes as a topic in undergraduate curricula might boost dental students and dentists' awareness of this device, given that our study showed a small percentage of participants had not received any formal information about dental loupes before.

The results of this study are important as it can be a cornerstone to reinforce stakeholders in dental clinic to provide dental loupes as safety measure for dental portioners. This can help them to reduce MSDs and help them to extend their career in healthy condition. Also, it is recommended that dental loupes be included as a topic in undergraduate curricula and that acquisition of dental loupes should be facilitated by subsidizing the costs, especially for dental students.

This study has some strengths, including the data being collected from four different dental colleges, including private ones, in Jeddah, and the use of the Nordic assessment as a validated tool. Among the limitations of the study, however, is that the study used a self-assessment questionnaire with a convenience sample. Also, because data were gathered from only Jeddah, the results do not have extremal validity for Saudi Arabia. It is recommended that a study be conducted to include many dental colleges around Saudi Arabia with a focus on only dental loupes.

## 5. Conclusion

There is a low occurrence of dental loupe use among dental students and dentists in Saudi Arabia. The use of dental loupes was associated with lower levels of MSDs in the lower back, neck, shoulders, elbows, upper back, and feet.

## Figures and Tables

**Table 1:** The study questionnaire.

Section	Question details
One	Questions about demographic data, including gender, age, academic year/work status, years of practice, financial status, and place of study/work.
Two	Questions asking about the participant's use of magnification devices (dental loupes, magnification glasses, and dental microscopes) and the sources of their knowledge about dental loupes.
Three	Attitude questions, including questions regarding which dental specialty should use dental loupes, the advantages and disadvantages of using loupes, and barriers to using dental loupes.
Four	The Nordic work-related MSD assessment [26], using selected items that investigated upper back, lower back, hip/thigh, neck, shoulder, elbow, wrist/hand, knee, and ankle/foot pain.

**Table 2:** Participant demographic data.

Variable	*N*	%
Gender		
Male	224	56.00
Female	176	44.00
Education/position		
2nd year	2	0.50
3rd year	5	1.30
4th year	7	1.80
5th year	40	10.00
6th year	79	19.80
Intern	150	37.50
General dentist	40	10.00
Postgraduate	6	1.50
Specialist	53	13.30
Consultant	18	4.50
College		
Private	277	69.25
Governmental	123	30.75

**Table 3:** Advantages and disadvantages of using dental loupes.

	*N*	%
*Advantages*		
Comfort in vision	237	59.25
Improvement in work accuracy	212	53.00
Improvement in quality of treatment	139	34.75
Time saving	104	26.00
Reduces muscle pain	88	22.00
None of the above	17	4.25

*Disadvantages*		
Dental loupes have no disadvantages	129	32.25
Difficulties in visual measurement	114	28.50
Pain in neck and shoulders	91	22.75
Low back pain	49	12.25
Becoming reliant on it	49	12.25
Pain in hands and wrists	41	10.25
Have not gotten around to using it	31	7.75

**Table 4:** Barriers to the use of dental loupes.

Barrier	*N*	%
Expensive	293	73.25
Better without magnification	39	9.75
Do not want to rely on dental loupes	32	8.00
Loupes make no difference in work	19	4.75
Used to use dental loupes but stopped due to health problems	7	1.75

**Table 5:** Participant prevalence of musculoskeletal pain related to dentistry.

Body part	Previous pain, ache, or discomfort, *N* (%)	Pain, ache, or discomfort in the past 7 days, *N* (%)
Lower back	254 (63.5%)	144 (36%)
Neck	261 (65.25%)	128 (32%)
Shoulder	185 (46.25%)	75 (18.75%)
Elbow	93 (23.25%)	31 (7.75%)
Wrist	133 (33.25%)	38 (9.5%)
Upper back	164 (41%)	82 (20.5%)
Hip	83 (20.75%)	44 (11%)
Knee	73 (18.25%)	49 (12.25%)
Feet	54 (13.5%)	32 (8%)

**Table 6:** The relationship between current and nonusers of dental loupes and a history of recent musculoskeletal trouble in the previous seven days.

Area	Pain	Currently a nonuser, *N* (%)	Current user of dental loupes, *N* (%)	*P* value
Lower back	Yes	137 (39.03)	7 (14.29)	0.001
	No	214 (60.97)	42 (85.71)	
Neck	Yes	119 (33.9)	9 (18.37)	0.029
	No	232 (66.1)	40 (81.63)	
Shoulder	Yes	75 (21.37)	0 (0)	<0.001
	No	276 (78.63)	49 (100)	
Elbow	Yes	31 (8.83)	0 (0)	0.022
	No	320 (91.17)	49 (100)	
Wrist	Yes	37 (10.54)	1 (2.04)	0.067
	No	314 (89.46)	48 (97.96)	
Upper back	Yes	80 (22.79)	2 (4.08)	0.001
	No	271 (77.21)	47 (95.92)	
Hip	Yes	42 (11.97)	2 (4.08)	0.141
	No	309 (88.03)	47 (95.92)	
Knee	Yes	47 (13.39)	2 (4.08)	0.065
	No	304 (86.61)	47 (95.92)	
Feet	Yes	32 (9.12)	0 (0)	0.022
	No	319 (90.88)	49 (100)	

## Data Availability

The data file of this study is available from the corresponding author upon reasonable request.
